# Comparing the clinical efficacy of hemoporfin-mediated photodynamic therapy for port wine stains in children with prior pulsed dye laser treatment history

**DOI:** 10.3389/fmed.2025.1667844

**Published:** 2025-11-21

**Authors:** Xiuwei Wang, Wei Su, Jianyou Chen, Yuhan Wang, Xiaoyan Liu, Gaolei Zhang, Sheng Zhang

**Affiliations:** 1 Translational Medicine Laboratory, Capital Center for Children's Health, Capital Medical University, Capital Institute of Pediatrics, Beijing, China; 2 Department of Dermatology, Capital Center for Children's Health, Capital Medical University, Beijing, China

**Keywords:** hemoporfin-photodynamic therapy, pulsed dye laser, efficacy, children, port wine stains, dermoscopy

## Abstract

**Background:**

Hemoporfin-mediated photodynamic therapy (HMME-PDT) is an effective and safe therapeutic modality for port wine stains (PWS). However, the clinical efficacy is influenced by multiple factors. This study aimed to evaluate the efficacy of HMME-PDT for children with PWS who have a prior history of pulsed dye laser (PDL) treatment.

**Methods:**

Clinical data were collected from children with PWS who received HMME-PDT treatment. The patients were divided into two groups: patients without PDL treatment history as the No PDL group and patients with a history of PDL as the Prior PDL group. The correlations between clinical efficacy and gender, age, lesion location, lesion color, dermoscopic vascular features, and number of treatments were analyzed in children with PWS.

**Results:**

A total of 216 patients were enrolled, including 100 patients in the No PDL group and 116 patients in the Prior PDL group aged between 1 and 14 years. There was no significant difference in clinical efficacy and adverse reactions of HMME-PDT treatment between the No PDL and Prior PDL groups. Both the location of lesions and dermoscopic vascular features significantly influenced the clinical efficacy of HMME-PDT for children with PWS (*p* < 0.05), while gender, age, lesion color, and number of treatments showed no association with the clinical efficacy. The location of lesions and dermoscopic vascular features might be an independent influencing factor for the clinical efficacy of HMME-PDT in children with PWS (*p* < 0.05).

**Conclusion:**

These data indicated that PDL treatment history did not influence the clinical efficacy of HMME-PDT and supported HMME-PDT as an alternative means to treat PDL-resistant PWS in children.

## 1 Introduction

Port wine stain (PWS) is a common capillary malformation, with an incidence of approximately 0.3–0.5% in newborns (1). The majority of PWS lesions are located on the face and neck and typically persist without spontaneous regression. Without timely and adequate intervention, these lesions progressively evolve from faint pink macules to thickened, nodular dark-red or purple plaques (2). In some patients, they may appear with varying degrees of disfigurement, which can negatively impact both their physical and psychological health (3). Consequently, developing effective therapeutic approaches to PWS remains critically important.

Pulsed dye laser (PDL) is considered “gold-standard” therapy for PWS, which acts through selective photothermolysis to eliminate the pathological blood vessels. However, clinical evidence indicates that fewer than 10% of patients achieve complete lesion clearance, and up to 20% of patients with PWS show poor outcomes after PDL treatment (4–6). Furthermore, emerging evidence suggests that the process of PDL treatment might promote neovascularization and cause the recurrence of PWS (7, 8). This unintended biological response potentially explains the observed high recurrence rates after PDL treatment of PWS. Considering the shortcomings of PDL, it is essential to explore more effective treatments for PWS.

Photodynamic therapy (PDT) is a relatively new modality used to treat both non-malignant and malignant diseases. In China, it is primarily used to treat PWS. This therapy utilizes photosensitizers combined with light to create a photochemical reaction in the target tissue, causing endothelial destruction and apoptosis. Hematoporphyrin monomethyl ether (HMME, also called hemoporfin), a second-generation photosensitizer, has been used in the treatment of PWS in China since 2017, after receiving approval from the China Food and Drug Administration (9). It demonstrates favorable pharmacokinetic properties, including reduced systemic toxicity, accelerated metabolic clearance, and enhanced photodynamic selectivity (10). HMME-mediated PDT (HMME-PDT) has emerged as an effective therapeutic approach for PWS, particularly in patients over 1 year of age who have large lesions or PDL resistance (11, 12). Currently, the modality is only available in China, and an expert consensus guideline was released last year (13).

Early intervention for PWS during infancy is recommended to obtain the optimal therapeutic outcomes. Comparative studies demonstrate that HMME-PDT is more effective in treating PWS than PDL (14). It is safe to use PDL treatment for PWS infants younger than 1 year (15). Meanwhile, there was little research on HMME-PDT for children with PWS under 1 year. Therefore, PDL treatment can effectively address the need for early intervention needs to impede the progression of PWS in young children who are unable to undergo HMME-PDT. Additionally, HMME-PDT provides a novel therapeutic alternative for children with PWS who show resistance to PDL treatment. However, there are some inconsistent conclusions regarding the impact of prior treatment history on the clinical efficacy of HMME-PDT. Studies showed that a greater number of prior PDL were associated with poor efficacy of HMME-PDT in PWS (16, 17). Wang et al. showed that previous PDL treatment history does not impact the effectiveness of HMME-PDT therapy in young children aged 1–3 years with PWS (18). In this study, we aim to evaluate the influence of previous PDL treatment on the efficacy of HMME-PDT in children with PWS. Or findings can provide valuable insights for optimizing HMME-PDT in children with PWS who are resistant to PDL treatment.

## 2 Materials and methods

### 2.1 Participant recruitment

Clinical data were collected from children with PWS who underwent HMME-PDT treatment at the Capital Center for Children’s Health, Capital Medical University, from January 2018 to December 2023. Patients with failure to follow up or accompanying other vascular malformations were excluded. This study was approved by the Ethics Committee of the Capital Center for Children’s Health, Capital Medical University, and was conducted in accordance with the Declaration of Helsinki. Informed consent was obtained from the patients’ guardians.

### 2.2 Treatment

HMME (5 mg/kg, Shanghai Fudan Zhangjiang Biomedical Co., Ltd., Shanghai, China) was administered intravenously at a consistent rate of 4 mL/min within 5 min. The lesions were exposed to a 532 nm LED light (LED Therapeutic Machine, LED-IE, Wuhan YaGe Optic and Electronic Technique Co. Ltd., Wuhan, Hubei, China) for 20 min, utilizing a power density range of 75–85 mW/cm^2^. The optical power meter (EPM-S700GWA4, Jiangsu Suzhou Aixin Electronic System Co., Ltd., China) was used to detect the output power during irradiation. The interval of HMME-PDT treatment was 2 to 3 months.

### 2.3 Image acquisition and evaluation

**Figure 1:**
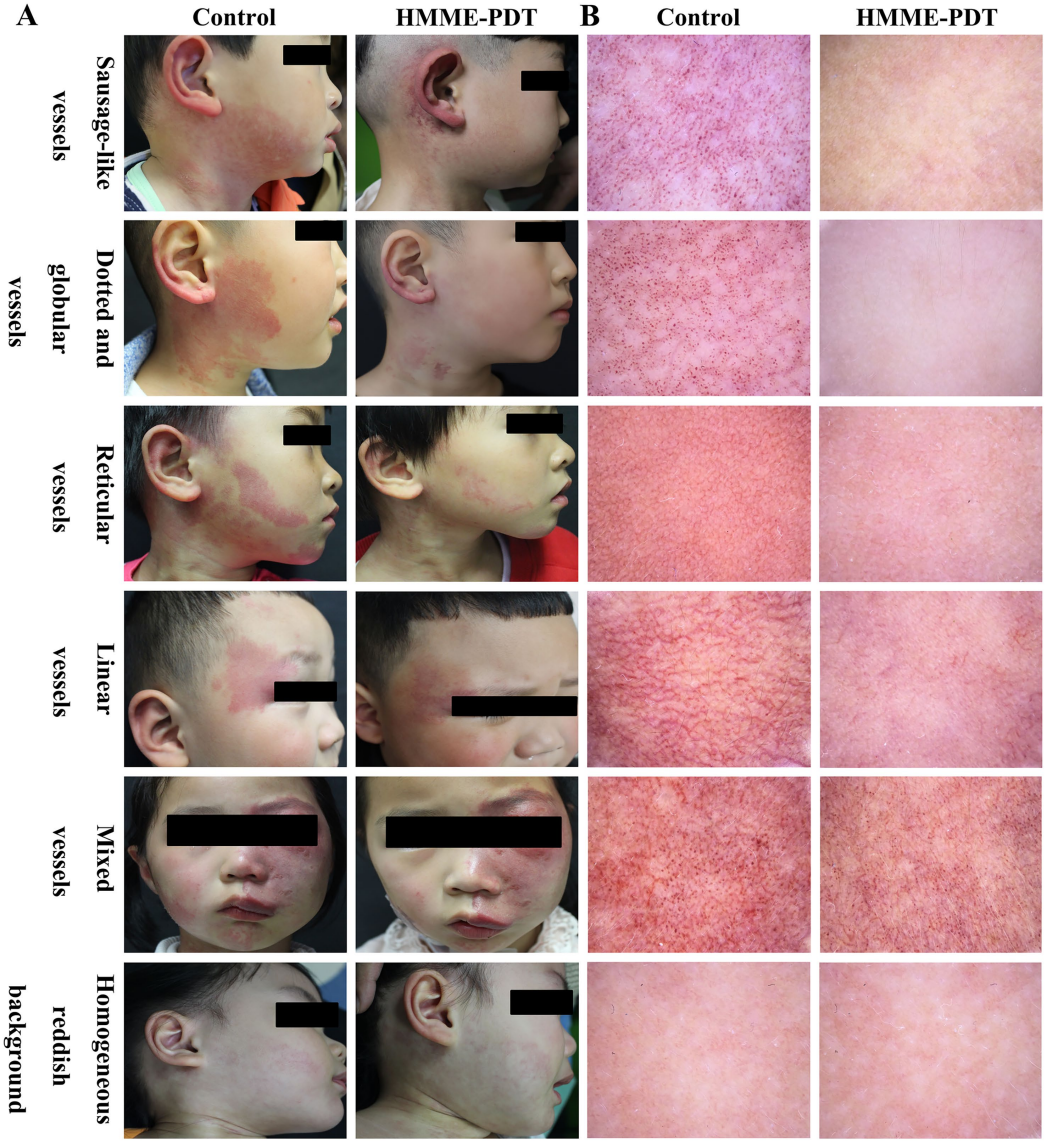
Clinical and dermoscopic features of lesions in children with PWS before and after HMME-PDT. **(A)** The clinical pictures of lesions in children with PWS before and after HMME-PDT. **(B)** The vascular features of lesions under dermoscope in children with PWS before and after HMME-PDT (×20).

A digital camera (Canon EOS700D, Canon, Japan) was applied to capture photographs of patients before and after HMME-PDT with uniform settings. Dermoscopic images were taken with a VEOS handheld dermatoscope (HD2, Canfield Scientific, United States). Only one area of the most clinically relevant lesions for each patient was included, and images from different fields were obtained for further analysis. Photographs or images were obtained by the same investigators. The vascular features were classified by three blinded dermatologists. If the vascular feature appearing in 80% of all images for a PWS were identified as the primary vascular feature. If the images included two or more vascular features in over 80%, the vascular feature was categorized as mixed. A non-vascular morphology was designated as a homogeneous reddish background. The dermoscopic vascular features were divided into sausage-like vessels, dot or globule vessels, reticular vessels, linear vessels, mixed vessels, and a homogeneous reddish background according to the previous study (19) (Figure 1).

### 2.4 Efficacy evaluation

Three blinded senior dermatologists independently evaluated the clinical efficacy according to the change in erythema before and after HMME-PDT treatment. The degree of lesion color blanching was assessed as follows: excellent (color blanching 75–100%), good (color blanching 50–74%), fair (color blanching 25–49%), and poor (color blanching < 25%). The findings were deemed valid when at least two out of three dermatologists concurred.

### 2.5 Safety analysis

Local or systemic adverse reactions such as pain, edema, purpura, scabs, infections, pigmentation, scar formation, and recurrence reported by patients or their guardians were recorded in detail both throughout the treatment and during the follow-up.

### 2.6 Statistical methods

The data analysis was performed using IBM SPSS Statistics Version 27.0, and the classified variables were expressed as frequencies (percentage). The chi-square test or Fisher’s exact test was utilized for the comparisons between the groups. The Mann–Whitney U-test was performed to compare efficacy between two independent samples. The Kruskal–Wallis test was used to assess overall efficacy and to conduct pairwise comparisons among multiple independent samples. All variables showing statistical significance in the univariate analysis were included in the multivariate logistic regression to assess independent outcome factors. Differences with a *p*-value of < 0.05 were considered statistically significant.

## 3 Results

### 3.1 Patients’ characteristics

A total of 216 patients with PWS were included in this study, including 100 (46.30%) patients with no prior PDL and 116 (53.70%) patients with prior PDL, who met the inclusion and exclusion criteria. The baseline characteristics of patients between the no PDL and prior PDL groups are shown in Table 1. The patients were predominantly 1–3 years of age and red for lesion color. There were no statistically significant differences in gender, age, lesion location, lesion color, vascular features, and number of treatments between the two groups (Table 1).

**Table 1:** General demographics of PWS patients.

Characteristic	*n*	No PDL, *n* (%)	Prior PDL, *n* (%)	*P*
Total	216	100 (46.30)	116 (53.70)	
Gender				
Male	108	52 (52.00)	56 (48.28)	0.585
Female	108	48 (48.00)	60 (51.72)	
Age (years)				
1–3	158	74 (74.00)	84 (72.41)	0.623
4–6	41	20 (20.00)	21 (18.10)	
7–14	17	6 (6.00)	11 (9.48)	
Location of lesions				
Central face	37	17 (17.00)	20 (17.24)	0.188
Peripheral face	75	36 (36.00)	39(33.62)	
Central and peripheral face	69	26 (26.00)	43 (37.07)	
Other locations	35	21 (21.00)	14 (12.07)	
Color of lesions				
Pink	38	16 (16.00)	22 (18.97)	0.850
Red	142	67 (67.00)	75 (64.65)	
Purple	36	17 (17.00)	19 (16.38)	
Vascular features				
Sausage-like vessels	44	21 (21.00)	23 (19.83)	0.521
Dotted and globular vessels	11	8 (8.00)	3 (2.59)	
Reticular vessels	47	20 (20.00)	27 (23.27)	
Linear vessels	63	26 (26.00)	37 (31.90)	
Mixed vessels	22	11 (11.00)	11 (9.48)	
Homogeneous reddish background	29	14 (14.00)	15 (12.93)	
Number of treatments				
3	76	42 (42.00)	34 (29.31)	0.051
≥4	140	58 (58.00)	82 (70.69)	

### 3.2 Efficacy of HMME-PDT in the treatment of PWS

Photographs of patients with PWS before and after HMME-PDT are shown in Figure 1. After HMME-PDT, a total of 81 (37.50%) patients showed excellent outcomes, 84 (38.89%) patients demonstrated good outcomes, 45 (20.83%) patients displayed fair outcomes, and 6 (2.78%) had poor outcomes. The rates of excellent outcomes were 41.00% in the No PDL group and 34.48% in the Prior PDL group, respectively. The efficacy of HMME-PDT showed no significant differences between the No PDL and Prior PDL groups (Table 2).

**Table 2:** Efficacy of HMME-PDT in the treatment of PWS between no PDL and prior PDL.

Group	*n*	Clinical efficacy *n* (%)				*Z*	*P*
		Excellent	Good	Fair	Poor		
Total	216	81 (37.50)	84 (38.89)	45 (20.83)	6 (2.78)		
No PDL	100	41 (41.00)	35 (30.00)	19 (19.00)	5 (5.00)	−0.482	0.630
Prior PDL	116	40 (34.48)	49 (42.24)	26 (22.41)	1 (0.86)		

### 3.3 Influencing factors of clinical efficacy in PWS patients

**Figure 2:**
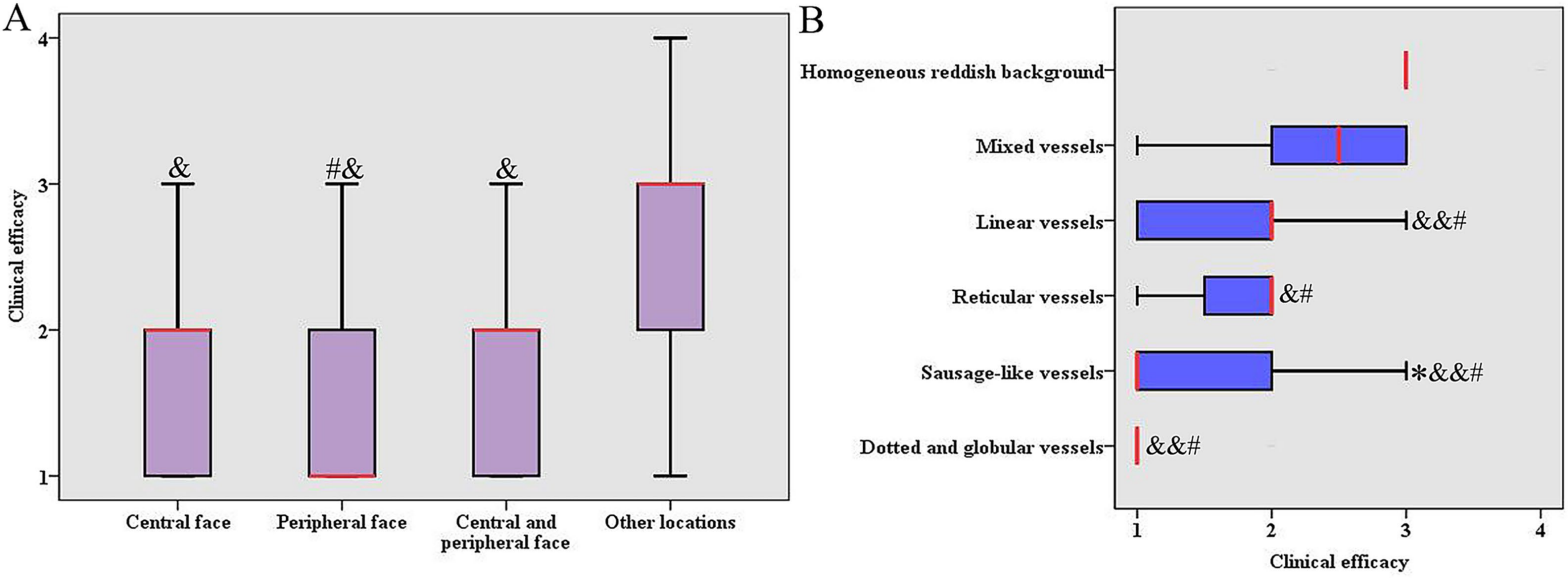
Comparing the clinical efficacy of HMME-PDT in different locations and vascular features. **(A)** Comparing the clinical efficacy of HMME-PDT in different locations. Y-axis “1” = excellent, “2” = improved, “3” = effective, “4” = poor. # *p* < 0.05 vs. central and peripheral face, and & *p* < 0.001 vs. other locations. **(B)** Comparing the clinical efficacy of HMME-PDT in different vascular features. X-axis “1” = excellent, “2” = improved, “3” = effective, “4” = poor. *p < 0.05 vs. reticular vessels, & *p* < 0.05 vs. mix vessels (more than 80% of the images exhibited two or more distinct vascular features), && *p* < 0.01 *vs.* mix vessels, # *p* < 0.001 vs. homogeneous reddish background (non-vascular morphology, representing a uniform red discoloration without visible individual vessels).

The influencing factors were performed with single-factor analysis for the clinical efficacy of HMME-PDT in 216 patients with PWS. The results showed that lesion locations and vascular features significantly influenced the clinical efficacy (*p* < 0.05), while gender, age, lesion color, and number of treatments did not show any statistically significant impact (*p* > 0.05, Table 3). Further analysis demonstrated that a notable difference was observed in the clinical efficacy of HMME-PDT for lesions located in the central face, peripheral face, and central and peripheral face compared to that in other locations (such as the trunk and limbs). The clinical efficacy of HMME-PDT was greater for lesions in the peripheral face than that for lesions in the central and peripheral face (*p* < 0.05, Figure 2A). Here, the vascular features under dermoscopic examination for 216 PWS were categorized into dot or globule vessels, sausage-like vessels, reticular vessels, linear vessels, mixed vessels, and homogeneous reddish background (Figure 1). Among the patients, 90.91% dotted and globular vessels and 72.73% with sausage-like vessels demonstrated excellent outcomes, respectively. Among the patients, 25.53% reticular vessels and 41.27% linear vessels exhibited excellent outcomes, respectively. A few excellent outcomes were observed in patients with mixed vessels or a homogeneous reddish background. The clinical efficacy of HMME-PDT was higher for vascular features, including dotted and globular vessels, sausage-like vessels, reticular vessels, and line vessels, compared to that for mixed vessels and, homogeneous reddish background. We also observed a higher efficacy in sausage-like vessels than in reticular vessels (*p* < 0.05, Figure 2B).

**Table 3:** Influencing factor of clinical efficacy in PWS patients with HMME-PDT.

Characteristic	*n*	Clinical efficacy n (%)				*P*
		Excellent	Good	Fair	Poor	
Gender						
Male	108	38 (35.19)	43 (39.81)	24 (22.22)	3 (2.78)	0.482
Female	108	43 (39.81)	41 (37.96)	21 (19.44)	3 (2.78)	
Age						
1–3	158	66 (41.77)	61 (38.61)	28 (17.72)	3 (1.90)	0.122
4–6	41	11 (26.83)	15 (36.58)	12 (29.27)	3 (7.32)	
7–14	17	4 (23.53)	8 (47.06)	5 (29.41)	0 (0.00)	
Lesion locations						
Central face	37	15 (40.54)	16 (43.24)	5 (13.51)	1 (2.70)	<0.001
Peripheral face	75	40 (53.33)	31 (41.33)	3 (4.00)	1 (1.33)	
Central and peripheral face	69	23 (33.33)	29 (42.03)	17 (24.64)	0 (0.00)	
Other locations	35	3 (8.57)	8 (22.86)	20 (57.14)	4 (11.43)	
Color of lesions						
Pink	38	11 (28.95)	16 (42.11)	8 (21.05)	3 (7.89)	0.386
Red	142	56 (39.44)	55 (38.73)	28 (19.72)	3 (2.11)	
Purple	36	14 (38.89)	13(36.11)	9 (25.00)	0 (0.00)	
Vascular features						
Dotted and globular vessels	11	10 (90.91)	1 (9.09)	0 (0.00)	0 (0.00)	<0.001
Sausage-like vessels	44	32 (72.73)	10 (22.73)	2 (4.54)	0 (0.00)	
Reticular vessels	47	12 (25.53)	33 (70.21)	2 (4.26)	0 (0.00)	
Linear vessels	63	26 (41.27)	29 (46.03)	8 (12.70)	0 (0.00)	
Mixed vessels	22	1 (4.55)	10 (45.45)	11 (50.00)	0 (0.00)	
Homogeneous reddish background	29	0 (0.00)	1 (3.45)	22 (75.86)	6 (20.69)	
Number of treatments						
3	76	25 (32.89)	32 (42.11)	16 (21.05)	3 (3.95)	0.356
≥4	140	56 (40.00)	52 (37.14)	29 (20.71)	3 (2.14)	

Multivariate logistic regression analysis showed that lesions located in the central face, peripheral face, and central and peripheral face, and different vascular features (dotted and globular vessels, sausage-like vessels, reticular vessels, linear vessels, and mixed vessels) emerged as independent influencing factors on the clinical efficacy of HMME-PDT (Supplementary Table 1).

To better determine the role of vascular features and lesion locations in the treatment of PWS with HMME-PDT, we determined the correlation between dermoscopic vascular features and lesion locations. The results showed that the dotted and globular vessels (54.5%) and sausage-like vessels (54.5%) were mainly located at the peripheral face, which was related to better outcomes. The homogeneous reddish background (51.7%) was located at other locations, which were associated with poor outcomes (Supplementary Table 2). These data indicate that different types of blood vessels in different locations may lead to different treatment outcomes.

### 3.4 Adverse reactions after HMME-PDT in children with PWS

Adverse reactions were observed, including pain, edema, purpura, scabby, itchy pigmentation, scar formation, and infection. There was no significant difference between the two groups in the occurrence of adverse reactions after HMME-PDT treatments (Table 4). All patients reported a burning sensation of varying intensity, which emerged within 2–4 min of starting the treatment and peaked at 6–10 min. The application of cold air circulation and cold compression could alleviate treatment-related discomfort, which disappeared on the same day. Edema and purpura were observed in all patients. Scabby (7.00%) and itching (1.00%) appeared, and pigmentation, scar formation, and infection were not observed in the no PDL group. Scabby (1.72%), itching (6.03%), pigmentation (0.86%), scar formation (1.72%), and infection (0.86%) were observed in the group with PDL prior to HMME-PDT. No other systemic adverse reactions were observed in the two groups. These results indicated that HMME-PDT is a safe treatment option for children with PWS.

**Table 4:** Adverse effects of HMME-PDT in the treatment of PWS between no PDL and prior PDL.

Evaluation indicators	*n*	No PDL (*n* = 100) *n* (%)	Prior PDL (116) *n* (%)	*P*
Edema	216	100 (100.00)	116 (100.00)	0.066
Purpura	216	100 (100.00)	116 (100.00)	
Scabby	9	7 (7.00)	2 (1.72)	
Itching	8	1 (1.00)	7 (6.03)	
Pigmentation	1	0 (0.00)	1 (0.86)	
Scar formation	2	0 (0.00)	2 (1.72)	
Infection	1	0 (0.00)	1 (0.86)	

Furthermore, in the follow-up after HMME-PDT, three patients showed recurrence in the regression area of lesions in the prior PDL group. None of the patients showed recurrence in patients without a PDL treatment history. The recurrence rates were 2.59% (3/116) for patients with prior PDL. Basic information regarding the recurrence of PWS after HMME-PDT is presented in Table 5. The ages of three patients showing recurrence were > 3 years. The lesions of color were purple, and the vascular features were mixed vessels. These patients received more than three sessions of HMME-PDT. The post-treatment outcomes were good and fair. The recurrence time was more than 30 months.

**Table 5:** Basic information of recurrence after HMME-PDT treatment in PWS.

No.	Gender	Age (years)	Lesion location	Times of prior PDL	Color	Vascular features	Efficacy	Times of HMME-PDT	Recurrence time (months)
1	Female	10	Peripheral face	2	Purple	Mixed	good	4	40
2	Female	6	Central and peripheral face	8	Purple	Mixed	fair	6	32
3	Male	11	Other locations	4	Purple	Mixed	fair	5	57

## 4 Discussion

Here, we retrospectively analyzed the clinical efficacy of HMME-PDT in 216 patients with PWS and evaluated the influencing factors of HMME-PDT on children with PWS. The results demonstrated that 81 (37.50%), 84 (38.89%), and 45 (20.83%) patients showed excellent, good, and fair outcomes. There were no significant differences in clinical efficacy between the No PDL and Prior PDL groups. Lesion locations and vascular features significantly influenced the clinical efficacy. No serious adverse effects were observed after the treatment between the two groups. These findings demonstrated that HMME-PDT is effective and safe for children with PWS, and PDL treatment history did not influence the clinical efficacy of HMME-PDT.

Given the thinner dermal structure, minimal pigment interference, and the risk of PWS progression (increased erythema thickness and surface hypertrophy) with age in pediatric patients, initiation of therapy during infancy is recommended (15, 20, 21). Early intervention for PWS is widely considered crucial for optimal therapeutic outcomes. At present, PDL is the gold standard for the treatment of PWS (7). Its well-documented safety can fulfill the early treatment for infants (0–1 year) with PWS. It operates on the principle of selective photothermolysis and targets hemoglobin within blood vessels, which absorbs the laser energy. This energy is converted to heat, denaturing the vessel wall proteins and damaging the endothelial cells. The resulting coagulation leads to thrombosis, occlusion, and eventual disappearance of the treated vessels, thereby achieving the therapeutic goal. However, it is constrained by low complete clearance rates, high recurrence, and frequent adverse reactions. Therefore, there is a pressing need to explore more effective therapeutic options for PWS. HMME-PDT, as the most promising strategy for the treatment of PWS, is viewed as more effective than PDL (22, 23), while its long-term impact on the developing central nervous system in young children remains unclear (24). There was little research on HMME-PDT for children with PWS under 1 year. Some children with PWS receiving HMME-PDT treatment have a prior history of PDL therapy. It is necessary to investigate whether PDL history affects the therapeutic efficacy of HMME-PDT. Several studies showed that a greater number of prior PDL were associated with poor efficacy of HMME-PDT in PWS (16, 17, 25). It was speculated that the occlusion of the ectatic vessels in PWS was replaced by fibrous connective tissue after laser treatment (26), which might reduce the light penetration and impair the efficacy of PDT. Furthermore, the superficial vessels were effectively targeted and eliminated by PDL, leaving only deeper, refractory vessels. However, Wang et al. showed that previous PDL treatment history does not impact the effectiveness of HMME-PDT therapy in young children aged 1–3 years with PWS (18), aligning with our findings of this study. It was speculated that prior PDL treatments may not increase the dermal fibrous connective tissue, thus not impairing the efficacy of HMME-PDT. Furthermore, it was reported that the coagulated vessels are below depths of 0.40 mm (mean 0.15 mm) by post-PDL biopsy for PWS (27). Studies suggest that for blood vessels with a diameter below 50 μm, the reduced hemoglobin concentration impedes laser energy absorption, rendering it insufficient to generate the thermal effect needed to destroy the vessel walls (6). Therefore, complete clearance of PWS is difficult to achieve by PDL. HMME-PDT is an effective alternative treatment for PWS. Utilizing photosensitizer HMME—a new generation of porphyrin photosensitizers—in conjunction with 532-nm laser-mediated PDT enables targeted capillary destruction with high penetration depth through intravascular photochemical and photothermal processes. Different photosensitizers possess distinct absorption bands. In general, the optimal laser wavelength for PDT is dictated by both the photosensitizer’s absorption profile and the pathological features of the disease. In China, HMME-PDT is primarily used for treating PWS, where the target vessels are in the superficial dermis. Although the primary absorption peak of HMME is approximately 400 nm (28), the limited tissue penetration at this wavelength is insufficient to reach the superficial dermis. Conversely, laser light approximately 630 nm, which corresponds to another absorption peak for HMME, can penetrate to the deep dermis, which risks damaging normal vasculature. Thus, the 532 nm laser wavelength is chosen to balance effective photosensitizer activation with targeted treatment depth for PWS. Han et al. suggested that vascular lesions within 800 μm of the dermal–epidermal junction were closed after PDT (532 nm laser light), and a stable clinical cure was achieved (29). In contrast to PDL, HMME-PDT could target capillaries of all diameters and downregulate the expression of vascular endothelial growth factor (VEGF). Some studies demonstrated that HMME-PDT has a positive effect on PDL-resistant PWS (16, 30). Here, we aimed to explore the influence of prior PDL on the clinical efficacy of HMME-PDT in children with PWS. Our results indicated that the prior PDL treatment history does not negatively affect the clinical efficacy of HMME-PDT in children with PWS, which provides helpful information with regard to the use of HMME-PDT for the treatment of PDL-resistant children with PWS.

The clinical efficacy of HMME-PDT is influenced by multiple factors. Here, we evaluated whether gender and age contributed to the efficacy of HMME-PDT. The results showed that gender and age do not affect the efficacy of HMME-PDT in children with PWS. Previous studies demonstrated that gender and age do not influence the efficacy of HMME-PDT for PWS (18, 31), aligning with our results. However, with advancing age, PWS lesions tend to darken, undergo hypertrophy and may develop nodules or a tendency to rupture. Thus, it was recommended that it be treated at an early age. Age is considered to be an important factor in the effectiveness of HMME-PDT treatment, and early intervention is recommended (32). Here, our findings may be attributed to slower disease progression and thinner dermal structures in children with PWS. Although there was no difference in overall treatment efficacy between Prior PDL and No PDL groups, a discrepancy was observed in the rate of excellent outcomes. Less epidermal melanin and dermal collagen, a thinner dermis, and lower blood volume in children contributed to better efficacy. Furthermore, given that HMME is administered based on the body weight of children with PWS, and considering that PWS lesions are smaller during childhood, the cost of treating the condition is relatively lower. Therefore, early intervention is recommended.

The anatomical location of PWS is a critical factor influencing the efficacy of HMME-PDT. Compared to other locations, such as the trunk and limbs, facial lesions had a better clinical efficacy. This is primarily due to the depth of blood vessels and the thickness of the skin. Regarding the facial lesions, the skin is thinner and the vessels are more superficial and smaller in diameter in the peripheral lesions than in the central lesions (30). Here, compared to other locations, the clinical efficacy of treatment for lesions located in the central face, the peripheral face, or both the central and peripheral face was higher. The clinical efficacy for lesions located in the peripheral face was higher than that in the central and peripheral face. Other locations (such as the trunk and limbs) are negatively factors for HMME-PDT treatment. It has been reported that the treatment outcomes were superior for lesions located in the peripheral face (including forehead, temples, cheeks, jaws, and chin) compared to those in the central face or the central and peripheral face (33); this trend is consistent with our study. This suggested that lesion location is closely related to the efficacy of HMME-PDT. The color of the lesion is another important factor in influencing the effectiveness of HMME-PDT. A previous study showed that pink lesions exhibit superior efficacy compared to purple lesions (18). In contrast to previous research, our study showed no significant difference in efficacy between pink, red, and purple lesions. That may be attributed to the thinner skin of children with PWS.

Vascular features of PWS classified by dermoscopy are probably the most important characteristics determining the clinical efficacy of PDL or HMME-PDT treatment. Dotted and globular vessels and sausage-like vessels belong to a superficial vascular pattern, and a 532-nm wavelength light source can reach these locations (34). Cases with sausage-like vessels, dotted and globular vessels showed better therapeutic effects (35). In the present study, sausage-like vessels and dotted and globular vessels showed high rates of excellent outcomes after treatment with HMME-PDT. Linear vessels and reticular vessels are deep vessels and are located on the reticular dermis (36). Previous studies demonstrated that the rate of excellent outcomes in cases with linear vessels and reticular vessels was significantly lower than that of sausage-like vessels, as well as dotted and globular vessels (37). Similar results were observed in our study. Furthermore, our study showed poor efficacy of HMME-PDT in mixed vessels and homogeneous reddish backgrounds, with low excellent rates. Previous studies showed that poor efficacy was observed after PDT treatment in patients with mixed and homogeneous reddish background vascular features (35), which is consistent with our results.

In general, the clinical efficacy of HMME-PDT was influenced by the number of treatment sessions (38). If the initial HMME-PDT session using conventional therapeutic parameters yields an inadequate response (color blanching <25%), the treatment parameters are adjusted based on the previous therapeutic response for two subsequent sessions. If the therapeutic response remains suboptimal, treatment discontinuation should be considered. In this study, patients underwent at least three sessions of HMME-PDT before evaluation of clinical efficacy. Although multiple sessions of HMME-PDT could cause better outcomes, the endpoint of treatment could not be clarified in this study. It is necessary to explore the treatment endpoints of HMME-PDT. Three patients showed recurrence in the regression area of lesions in the prior PDL group. The recurrence of PWS might be associated with the induction of angiogenesis and the revascularization of lesions after PDL treatment. Furthermore, mixed vessels are located in the lower and middle dermis and have larger diameters, thicker walls, and denser collagen fibers, which could impede the penetration of the light source and impair the efficacy of PDT for PWS. Our study showed poor efficacy of HMME-PDT in mixed vessels, with an excellent rate of 4.55%. The gene GNAQ p. R183Q mutation is more common in patients with PWS (39). The mutation could have heightened resistance to HMME-PDT-induced apoptosis (40). Based on the above-mentioned, the residual angiogenic activity persisted following HMME-PDT treatment, finally leading to the recurrence of lesions in PWS.

There are several limitations to our study. It was a single-center analysis, and the sample size was small. Secondly, this was a retrospective study, which lacked images of patients prior to PDL treatment, which influenced the evaluation of clinical outcomes. Furthermore, detailed information about PDL treatment and its implications for evaluation were not provided due to the patient having undergone PDL treatment at a different clinic before enrollment in this study. Therefore, multicenter, large-sample, randomized controlled studies are required to provide more reliable evidence.

## 5 Conclusion

In summary, previous PDL treatment history had no significant impact on the clinical efficacy of HMME-PDT in pediatric patients with PWS. These findings support the use of HMME-PDT as an alternative means of treating PDL-resistant PWS in children. The lesion locations and dermoscopic vascular features were closely related to the clinical outcomes of HMME-PDT. Vascular features with sausage-like vessels and dotted and globular vessels, and lesions located in the peripheral face, showed better efficacy in PWS, which indicated that vascular features and lesion locations may be indicators for predicting and evaluating the clinical efficacy of HMME-PDT for PWS in children.

## Data Availability

The raw data supporting the conclusions of this article is available from the corresponding author on reasonable request.

## References

[1] Jacobs AH Walton RG. The incidence of birthmarks in the neonate. Pediatrics. (1976) 58:218–22. doi: 10.1542/peds.58.2.218 951136

[2] Updyke KM Khachemoune A. Port-wine stains: a focused review on their management. J Drugs Dermatol. (2017) 16:1145–51. Available online at: https://pubmed.ncbi.nlm.nih.gov/29141064/ PMID: 29141064

[3] Hagen SL Grey KR Korta DZ Kelly KM. Quality of life in adults with facial port-wine stains. J Am Acad Dermatol. (2017) 76:695–702. doi: 10.1016/j.jaad.2016.10.039, PMID: 27955934 PMC5790427

[4] Huikeshoven M Koster PH de Borgie CA Beek JF van Gemert MJ van der Horst CM. Redarkening of port-wine stains 10 years after pulsed-dye-laser treatment. N Engl J Med. (2007) 356:1235–40. doi: 10.1056/NEJMoa064329, PMID: 17377161

[5] Savas JA Ledon JA Franca K Chacon A Nouri K. Pulsed dye laser-resistant port-wine stains: mechanisms of resistance and implications for treatment. Br J Dermatol. (2013) 168:941–53. doi: 10.1111/bjd.12204, PMID: 23290045

[6] Jasim ZF Handley JM. Treatment of pulsed dye laser-resistant port wine stain birthmarks. J Am Acad Dermatol. (2007) 57:677–82. doi: 10.1016/j.jaad.2007.01.019, PMID: 17658196

[7] Wang B Mei X Wang Y Hu X Li F. Adjuncts to pulsed dye laser for treatment of port wine stains: a literature review. J Cosmet Laser Ther. (2021) 23:209–17. doi: 10.1080/14764172.2022.2052901, PMID: 35422188

[8] Musalem HM Alshaikh AA Tuleimat LM Alajlan S. Outcome with topical sirolimus for port wine stain malformations after unsatisfactory results with pulse dye laser treatment alone. Ann Saudi Med. (2018) 38:376–80. doi: 10.5144/0256-4947.2018.376, PMID: 30284993 PMC6180221

[9] Zhang Y Zou X Chen H Yang Y Lin H Guo X. Clinical study on clinical operation and post-treatment reactions of HMME-PDT in treatment of PWS. Photodiagn Photodyn Ther. (2017) 20:253–6. doi: 10.1016/j.pdpdt.2017.09.013, PMID: 29079350

[10] Pu Y Chen W Yu Z. Research progress of Hemoporfin – part one: preclinical study. Photodiagn Photodyn Ther. (2012) 9:180–5. doi: 10.1016/j.pdpdt.2011.09.004, PMID: 22594989

[11] Chun-Hua T Li-Qiang G Hua W Jian Z Si-Li N Li L . Efficacy and safety of hemoporfin photodynamic therapy for port-wine stains in paediatric patients: a retrospective study of 439 cases at a single Centre. Photodiagn Photodyn Ther. (2021) 36:102568. doi: 10.1016/j.pdpdt.2021.102568, PMID: 34614424

[12] Han Y Ying H Zhang X Yu W Cen Q Chen X . Retrospective study of photodynamic therapy for pulsed dye laser-resistant port-wine stains. J Dermatol. (2020) 47:348–55. doi: 10.1111/1346-8138.15238, PMID: 32012364

[13] Consensus development expert group of hematoporphyrin monomethyl ether-mediated photodynamic therapy for treating port-wine stains. Expert consensus on hematoporphyrin monomethyl ether-mediated photodynamic therapy for treating port-wine stains. Chin J Dermatol. (2024) 57:581–8. doi: 10.35541/cjd.20230584 (Chinese)

[14] Zhang B Zhang TH Huang Z Li Q Yuan KH Hu ZQ. Comparison of pulsed dye laser (PDL) and photodynamic therapy (PDT) for treatment of facial port-wine stain (PWS) birthmarks in pediatric patients. Photodiagn Photodyn Ther. (2014) 11:491–7. doi: 10.1016/j.pdpdt.2014.06.004, PMID: 24973576

[15] Jeon H Bernstein LJ Belkin DA Ghalili S Geronemus RG. Pulsed dye laser treatment of port-wine stains in infancy without the need for general anesthesia. JAMA Dermatol. (2019) 155:435–41. doi: 10.1001/jamadermatol.2018.5249, PMID: 30865245 PMC6459097

[16] Lv Y Yang L Wei S Xiangli X Yang X Zhang X . Possible number of recommended sessions and influential factors of Hematoporphyrin monomethyl ether photodynamic therapy for pulsed dye laser-resistant facial port-wine stain. Lasers Surg Med. (2025) 57:312–20. doi: 10.1002/lsm.70003, PMID: 40098285

[17] Huang Y Yang J Sun L Zhang L Bi M. Efficacy of influential factors in hemoporfin-mediated photodynamic therapy for facial port-wine stains. J Dermatol. (2021) 48:1700–8. doi: 10.1111/1346-8138.16094, PMID: 34355416

[18] Wang Y Zhou J Qi C Li Y Hu D Zhang R . Evaluation of efficacy of Hemoporfin-PDT in the treatment of PWS birthmarks of young children previously received PDL treatment. Photodiagn Photodyn Ther. (2024) 50:104418. doi: 10.1016/j.pdpdt.2024.104418, PMID: 39592021

[19] Zhang S Wang X Chen Y Wang Y Chen J Liu X . Retrospective analysis of the correlation between dermoscopic vascular features and the response to hemoporfin-mediated photodynamic therapy in previously untreated children with port wine stains. Photodiagn Photodyn Ther. (2025) 54:104691. doi: 10.1016/j.pdpdt.2025.104691, PMID: 40578726

[20] Brightman LA Geronemus RG Reddy KK. Laser treatment of port-wine stains. Clin Cosmet Investig Dermatol. (2015) 8:27–33. doi: 10.2147/CCID.S53118, PMID: 25624768 PMC4296879

[21] Ashinoff R Geronemus RG. Flashlamp-pumped pulsed dye laser for port-wine stains in infancy: earlier versus later treatment. J Am Acad Dermatol. (1991) 24:467–72. doi: 10.1016/0190-9622(91)70075-D, PMID: 2061448

[22] Gao K Huang Z Yuan KH Zhang B Hu ZQ. Side-by-side comparison of photodynamic therapy and pulsed-dye laser treatment of port-wine stain birthmarks. Br J Dermatol. (2013) 168:1040–6. doi: 10.1111/bjd.12130, PMID: 23137063

[23] Peng X Ye T Yu B Liu X Liu L. Comparing HMME-PDT and Cynergy dual-wavelength laser in the treatment of facial PWS. Photodiagn Photodyn Ther. (2022) 37:102703. doi: 10.1016/j.pdpdt.2021.102703, PMID: 34965475

[24] Gao C Nguyen V Hochman ML Gao L Chen EH Friedman HI . Current clinical evidence is insufficient to support HMME-PDT as the first choice of treatment for young children with port wine birthmarks. Lasers Surg Med. (2024) 56:321–33. doi: 10.1002/lsm.23779, PMID: 38506454 PMC12422306

[25] Chen J Gui Y Wang S Huang D Lyu J Cheng H . Analysis of related factors affecting hemoporfin-mediated photodynamic therapy for port-wine stain: a retrospective study. Photodermatol Photoimmunol Photomed. (2023) 39:441–8. doi: 10.1111/phpp.12874, PMID: 37036012

[26] Walker EP Butler PH Pickering JW Day WA Fraser R Van Halewyn CN. Histology of port wine stains after copper vapour laser treatment. Br J Dermatol. (1989) 121:217–23. doi: 10.1111/j.1365-2133.1989.tb01801.x, PMID: 2775646

[27] Fiskerstrand EJ Svaasand LO Kopstad G Ryggen K Aase S. Photothermally induced vessel-wall necrosis after pulsed dye laser treatment: lack of response in port-wine stains with small sized or deeply located vessels. J Invest Dermatol. (1996) 107:671–5. doi: 10.1111/1523-1747.ep12365566, PMID: 8875947

[28] Li BH Xie SC Lu ZK. Spectral properties of new photosensitizers for photodynamic diagnosis and therapy. Spectrosc Spectr Anal. (2002) 22:902–4. doi: 10.3321/j.issn:1000-0593.2002.06.008 (Chinese) 12914159

[29] Han Y Yu W Wang L Cen Q Luo L Zhu J . Histological characteristics of port-wine stains with complete regression after photodynamic therapy treatment: a 7-year follow-up. Photobiomodul Photomed Laser Surg. (2022) 40:159–62. doi: 10.1089/photob.2021.0111, PMID: 35298284

[30] Zhang LC Yang J Huang YB Bi MY. Efficacy of hemoporfin photodynamic therapy for pulsed dye laser-resistant facial port-wine stains in 107 children: a retrospective study. Indian J Dermatol Venereol Leprol. (2022) 88:275. doi: 10.25259/IJDVL_976_20, PMID: 34672476

[31] Liu J Zhou J Hu D Cui L Li Y Ye D . Retrospective analysis of Hemoporfin-mediated photodynamic therapy in the treatment of naive port-wine stains. Photodiagn Photodyn Ther. (2022) 39:103003. doi: 10.1016/j.pdpdt.2022.103003, PMID: 35840007

[32] Li-Qiang G Hua W Si-Li N Chun-Hua T. A clinical study of HMME-PDT therapy in Chinese pediatric patients with port-wine stain. Photodiagn Photodyn Ther. (2018) 23:102–5. doi: 10.1016/j.pdpdt.2018.06.006, PMID: 29885812

[33] Nguyen CM Yohn JJ Huff C Weston WL Morelli JG. Facial port wine stains in childhood: prediction of the rate of improvement as a function of the age of the patient, size and location of the port wine stain and the number of treatments with the pulsed dye (585 nm) laser. Br J Dermatol. (1998) 138:821–5. doi: 10.1046/j.1365-2133.1998.02219.x, PMID: 9666828

[34] Kwiek B Rozalski M Sieczych J Paluch L Kowalewski C Ambroziak M. Predictive value of dermoscopy for the treatment of port-wine stains with large spot 532 nm laser. Lasers Surg Med. (2019) 51:569–83. doi: 10.1002/lsm.23083, PMID: 30860283

[35] Wang X Suo H Gao Y Du H Fu Y Sha S . Correlation between the hemoporfin-mediated photodynamic treatment response and the dermoscopy vascular pattern in patients with a port-wine stain: a prospective study. J Eur Acad Dermatol Venereol. (2020) 34:2795–801. doi: 10.1111/jdv.16596, PMID: 32401355

[36] Sevila A Nagore E Botella-Estrada R Sanmartin O Requena C Serra-Guillen C . Videomicroscopy of venular malformations (port-wine stain type): prediction of response to pulsed dye laser. Pediatr Dermatol. (2004) 21:589–96. doi: 10.1111/j.0736-8046.2004.21514.x, PMID: 15461769

[37] Eubanks LE McBurney EI. Videomicroscopy of port-wine stains: correlation of location and depth of lesion. J Am Acad Dermatol. (2001) 44:948–51. doi: 10.1067/mjd.2001.113467, PMID: 11369905

[38] Kang J Liu JJ Fang YH Lin YY Gong W Wang HY . Hemoporfin-mediated photodynamic therapy for port-wine stains on extremities. Dermatol Ther (Heidelb). (2023) 13:1857–71. doi: 10.1007/s13555-023-00970-8, PMID: 37405633 PMC10366063

[39] Liu L Li X Zhao Q Yang L Jiang X. Pathogenesis of port-wine stains: directions for future therapies. Int J Mol Sci. (2022) 23:12139. doi: 10.3390/ijms232012139, PMID: 36292993 PMC9603382

[40] Liu L Wang L Luo J Yu J Xie L Liu Y . Endothelial GNAQ p.R183Q mutation confers hemoporfin-mediated photodynamic therapy resistance and drives pathological angiogenesis via the angiopoietin-2/TIE2/PI3K/AKT pathway. Front cell. Dev Biol. (2025) 13:1622961. doi: 10.3389/fcell.2025.1622961, PMID: 40917747 PMC12411732

