# Supplementary material for: Comparing the clinical efficacy of hemoporfin-mediated photodynamic therapy for port wine stains in children with prior pulsed dye laser treatment history
Source: Front Med (Lausanne). 2025 Nov 21;12:1667844. doi: 10.3389/fmed.2025.1667844 (PMC12678093; doi:10.3389/fmed.2025.1667844)
Supplement: Supplementary file 1 [file Table_1.docx]

**Supplemental Table 1** **Multiple-factor logistic regression analysis of the clinical efficacy of HMME-PDT.**

|  | **Estimate** | **Standard error** | **Wald** | **Freedom** | **Significance** |
| --- | --- | --- | --- | --- | --- |
| Central face | -1.242 | 0.561 | 4.893 | 1 | 0.027 |
| Peripheral face | -1.985 | 0.532 | 13.928 | 1 | 0.000 |
| Central and peripheral face | -1.787 | 0.511 | 12.230 | 1 | 0.000 |
| Other locations | 0 | . | . | 0 | . |
| Dotted and globular vessels | -8.642 | 1.523 | 32.205 | 1 | 0.000 |
| Sausage-like vessels | -6.956 | 1.117 | 38.802 | 1 | 0.000 |
| Reticular vessels | -5.307 | 1.083 | 24.003 | 1 | 0.000 |
| Linear vessels | -5.811 | 1.074 | 29.260 | 1 | 0.000 |
| Mixed vessels | -3.238 | 1.088 | 8.855 | 1 | 0.003 |
| Homogeneous reddish background | 0 | . | . | 0 | . |

Supplemental Table 2 **Correlation of dermoscopic vascular feature with lesion locations**

| **Vascular features** | **Peripheral face** | **Central face** | **Central and peripheral face** | **Other locations** |
| --- | --- | --- | --- | --- |
| Sausage-like vessels n=44 | 24 (54.5) | 8 (18.2) | 12 (27.3) | 0 (0.0) |
| Dotted and globular vessels n=11 | 6 (54.5) | 2 (18.2) | 2 (18.2) | 1 (9.1) |
| Reticular vessels n=47 | 22 (46.8) | 9 (19.1) | 12 (25.5) | 4 (8.5) |
| Linear vessels n=63 | 17 (27.0) | 15 (23.8) | 20 (31.7) | 11 (17.5) |
| Mixed vessels n=22 | 4 (18.2) | 2 (9.1) | 12(54.5) | 4 (18.2) |
| Homogeneous reddish background n=29 | 2 (6.9) | 1 (3.4) | 11 (37.9) | 15 (51.7) |
